# Supplementary material for: Contemporary climatic analogs for 540 North American urban areas in the late 21st century
Source: Nat Commun. 2019 Feb 12;10:614. doi: 10.1038/s41467-019-08540-3 (PMC6372656; doi:10.1038/s41467-019-08540-3)
Supplement: Supplementary file 1 — Supplementary Information [file 41467_2019_8540_MOESM1_ESM.pdf]

Supplementary Information for:

Contemporary climatic analogs for 540 North American urban areas in the late 21st century

Fitzpatrick et al.

Supplementary Figures

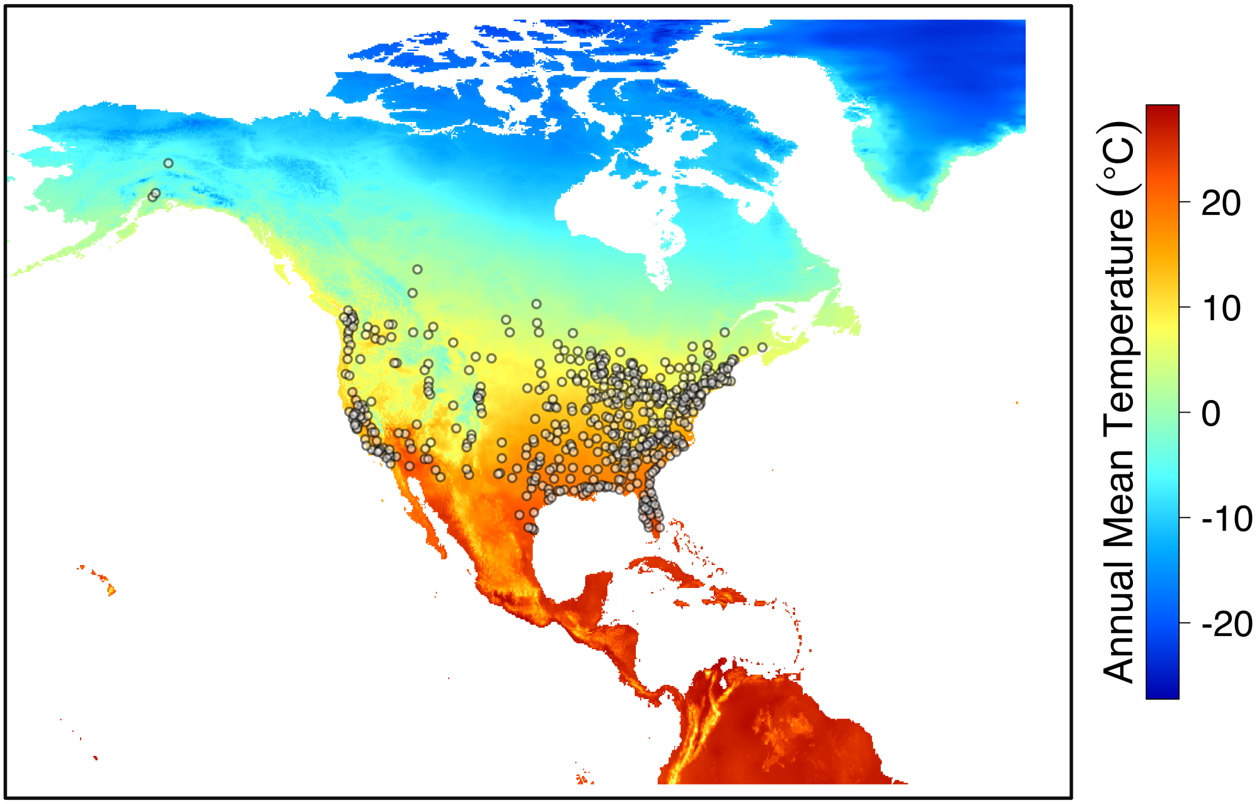

Supplementary Figure 1 | Extent of the study domain and locations of urban areas. Shading shows the gradient of annual mean temperature representative of the period 1960-1990 at 5 arc-minute resolution from the WorldClim dataset[1]. Points indicate grid cells containing an urban area for which climatic analogs were quantified. Note that in order to improve readability of figures, results for urban areas in Alaska are not reported in the manuscript but are available online at https://tinyurl.com/urbanclimate.

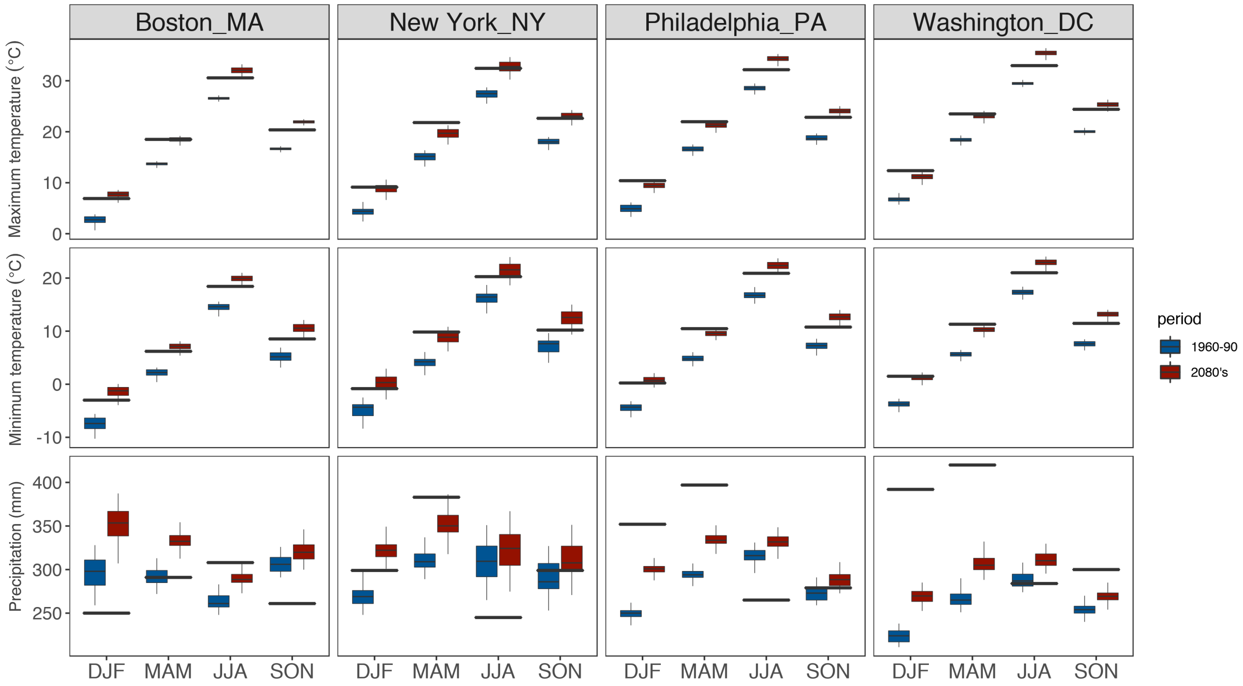

Supplementary Figure 2 | Contemporary and future climate versus best climatic analog. Boxplots show variability (median, first and third quartiles, and the inter-quartile range × 1.5) of contemporary (1960-1990) and future climate (2080's, ensemble mean of RCP8.5 scenarios for the period 2070-2099) within each of four eastern North America cities for the four climatological seasons. Bold lines indicate climatic conditions at the geographic location identified as the best contemporary climatic analog for that city and the ensemble mean of RCP8.5 scenarios. DJF=winter, MAM=spring, JJA=summer, SON=autumn.

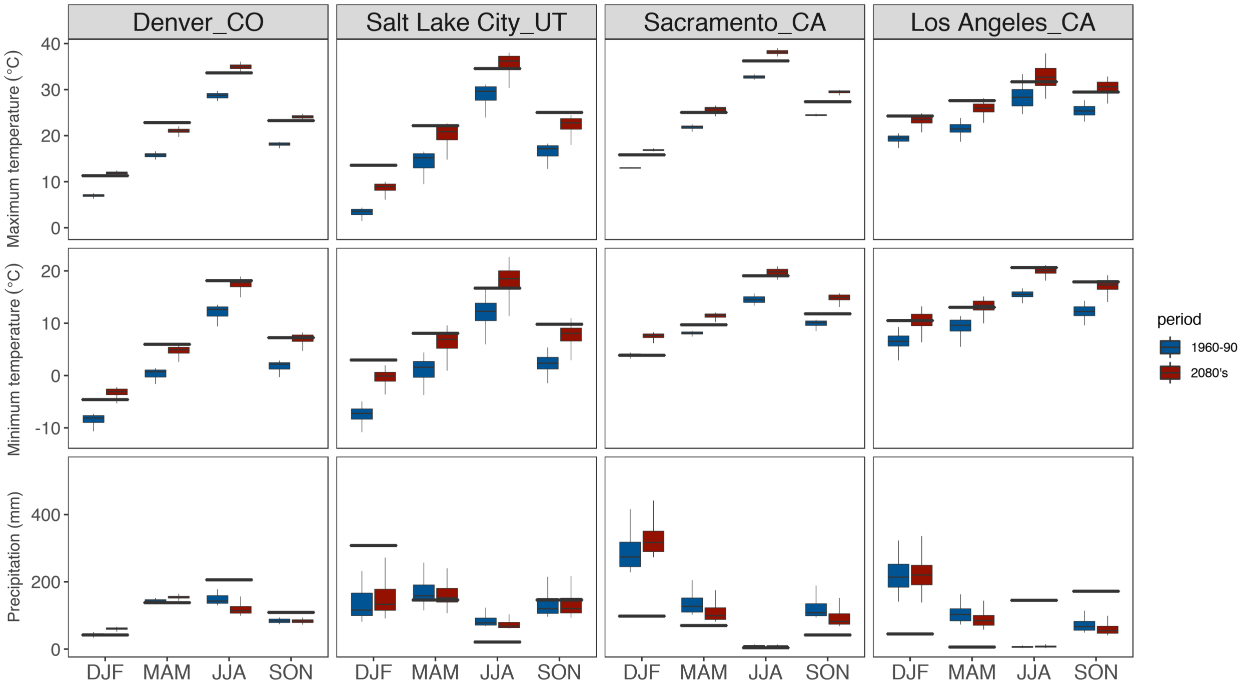

Supplementary Figure 3 | Contemporary and future climate versus best climatic analog. Boxplots show variability (median, first and third quartiles, and the inter-quartile range × 1.5) of contemporary (1960-1990) and future climate (2080's, ensemble mean of RCP8.5 scenarios for the period 2070-2099) within each of four western North America cities for the four climatological seasons. Bold lines indicate climatic conditions at the geographic location identified as the best contemporary climatic analog for that city and the ensemble mean of RCP8.5 scenarios. DJF=winter, MAM=spring, JJA=summer, SON=autumn.

Supplementary Tables

Supplementary Table 1 | Earth system models used for future climate projections. All 27 models produced projections for both RCP4.5 and RCP8.5 for the period 2070-2099. The CCFAS ID column refers to how the data are identified by the Consultative Group for International Agricultural Research (CGIAR) Research Program on Climate Change, Agriculture and Food Security (CCAFS; http://www.ccafs-climate.org/)[2].

Modeling Center	Model	CCAFS ID
BCC	BCC-CSM1.1	bcc_csm1_1
	BCC-CSM1.1(m)	bcc_csm1_1_m
GCESS	BNU-ESM	bnu_esm
CCCma	CanESM2	cccma_canesm2
NSF-DOE-NCAR	CESM1(BGC)	cesm1_bgc
	CESM1(CAM5)	cesm1_cam5
CSIRO-BOM	ACCESS1.0	csiro_access1_0
	ACCESS1.3	csiro_access1_3
CSIRO-QCCCE	CSIRO-Mk3.6.0	csiro_mk3_6_0
FIO	FIO-ESM	fio_esm
NOAA GFDL	GFDL-ESM2G	gfdl_esm2g
	GFDL-ESM2M	gfdl_esm2m
NASA GISS	GISS-E2-R	giss_e2_r
INM	INM-CM4	inm_cm4
IPSL	IPSL-CM5A-LR	ipsl_cm5a_lr
	IPSL-CM5A-MR	ipsl_cm5a_mr
LASG-CESS	FGOALS-g2	lasg_fgoals_g2
MIROC	MIROC-ESM-CHEM	miroc_esm_chem
	MIROC-ESM	miroc_esm
	MIROC5	miroc_miroc5
MOHC	HadGEM2-CC	mohc_hadgem2_cc
	HadGEM2-ES	mohc_hadgem2_es
MPI-M	MPI-ESM-LR	mpi_esm_lr
MRI	MRI-CGCM3	mri_cgcm3
NCAR	CCSM4	ncar_ccsm4
NCC	NorESM1-M	ncc_noresm1_m
NIMR/KMA	HadGEM2-AO	nimr_hadgem2_ao

Supplementary References

1. Hijmans, R. J., Cameron, S. E., Parra, J. L., Jones, P. G. & Jarvis, A. Very high resolution interpolated climate surfaces for global land areas. *Int. J. Climatol.* **25**, 1965–1978 (2005).

2. CGIAR Research Program on Climate Change, Agriculture and Food Security. doi:10.1163/9789004322714_cclc_2015-0171-002
